# Efficacy of Ronopterin (VAS203) in Patients with Moderate and Severe Traumatic Brain Injury (NOSTRA phase III trial): study protocol of a confirmatory, placebo-controlled, randomised, double blind, multi-centre study

**DOI:** 10.1186/s13063-019-3965-4

**Published:** 2020-01-14

**Authors:** Frank Tegtmeier, Reinhard Schinzel, Ronny Beer, Diederik Bulters, Jean-Yves LeFrant, Joan Sahuquillo, Andreas Unterberg, Peter Andrews, Antonio Belli, Javier Ibanez, Alfonso Lagares, Michael Mokry, Harald Willschke, Charlotte Flühe, Erich Schmutzhard, on behalf of the NOSTRA Investigators

**Affiliations:** 1 vasopharm GmbH, Würzburg, Germany; 2 Medizinische Universität Innsbruck, Innsbruck, Austria; 3 Wessex Neurological Centre University Hospital, Southampton, UK; 4 Hopital Universitaire Caremeau , Nimes, France; 5 Vall d’Hebron University Hospital , Barcelona, Spain; 6 Universitätsklinikum Heidelberg, Heidelberg, Germany; 7 Western General Hospital Lothian University , Edinburgh, UK; 8 Queen Elizabeth Hospital, Birmingham, UK; 9 Espases University Hospital , Palma de Mallorca, Spain; 10 Hospital Universitario 12 de Octubre , Madrid, Spain; 11 LKH – Universitätsklinikum Graz, Graz, Austria; 12 Medizinische Universität Wien, Wien, Austria; 13 Universitätsklinikum Schleswig-Holstein, Kiel, Germany

**Keywords:** Traumatic brain injury, Outcome, Nitric oxide synthase inhibition, Randomised controlled trials

## Abstract

**Background:**

Traumatic brain injury is a leading cause of death and disability worldwide. The nitric oxide synthase inhibitor Ronopterin was shown to improve clinical outcome by enhancing neuroprotection in a phase IIa trial.

**Methods/design:**

The NOSTRA phase III trial (Ronopterin in traumatic brain injury) is a multi-centre, prospective, randomised, double-blinded, placebo-controlled, phase III trial in Europe. It aims at determining whether the administration of Ronopterin compared to placebo improves neurological outcome in patients with moderate or severe traumatic brain injury at 6 months after injury. The trial is designed to recruit patients between 18 and 60 years of age with moderate or severe traumatic brain injury (Glasgow Coma Scale score ≥ 3) and requiring insertion of an intracranial pressure probe. Trial patients will receive a 48-h intravenous infusion of either Ronopterin or placebo starting at the earliest 6 h and at the latest 18 h after injury. The primary outcome will be the extended Glasgow Outcome Score (eGOS) at 6 months. Secondary outcomes will include the Quality of Life Index (QOLIBRI) at 6 months after the injury and the eGOS at 3 months after the injury. Additionally, effects on mortality, intracranial pressure and cerebral perfusion pressure are evaluated.

**Discussion:**

The trial aims to provide evidence on the efficacy and safety of Ronopterin in patients with traumatic brain injury.

**Trial registration:**

EudraCT, 2013–003368-29. Registered on 9 March 2016.

ClinicalTrials.gov, NCT02794168. Registered on 8 June 2016.

Protocol version 14.0 from 05 November 2018.

## Background

Traumatic brain injury (TBI) is a major cause of mortality and long-term disability, with enormous impact on patients and their families [1]. In Europe an overall incidence rate of 262 per 100,000 for patients admitted with TBI was reported in a meta-analysis [2]. The TBI related costs are high and account for €33 billion in Europe in 2010. The high costs are owed mostly to lifetime productivity losses, particularly when young people are affected [3].

Clinical trials in TBI with pharmacological interventions have failed so far, most likely due to the heterogeneity of the disease and its treatment [4]. The pathophysiology of TBI is complex and involves a variety of processes including - among others - neuroinflammation, brain oedema formation and excitotoxicity. Nitric oxide has been discussed as key player in the development of secondary injury after TBI [5]. Inhibitors of nitric oxide synthase have been tested in animal models of TBI [6]. Due to their unique properties, co-factor analogues such as 4-amino-tetrahydrobiopterin (Ronopterin, VAS203), the anti-pterins, have been found to be particularly useful in animal models of TBI [7, 8].

The safety and pharmacodynamics of Ronopterin were assessed in TBI in an exploratory randomised, placebo-controlled and blinded phase II study (“NO synthase inhibition in traumatic brain injury” (NOSTRA)). In an exploratory analysis the study showed promising results by significantly improving clinical outcome despite the small number of patients enrolled [9]. Ronopterin was found to be safe in general; however, renal failure was observed in the highest-dose group, and this was possibly related to Ronopterin. The mechanism of Ronopterin in kidney function was investigated in healthy volunteers, showing a reversible pharmacodynamic inhibitory effect of Ronopterin on renal plasma flow [10].

The ongoing NOSTRA phase III study is a European multi-centre, blinded, randomised, parallel group, placebo-controlled, phase III trial of Ronopterin administration in adults (age 18–60 years) with acute TBI of moderate or greater severity. Based on the results of the NOSTRA phase II study, the trial is designed to detect clinically relevant differences in clinical outcome (extended Glasgow Outcome Score (Egos) at 6 months after injury) [11] as the primary endpoint.

## Methods/design

### Trial design

NOSTRA-III is a multi-centre, prospective, parallel-group (two groups), blinded, placebo-controlled, randomised, phase III trial of Ronopterin administration to adults with TBI of moderate or greater severity requiring intensive care. The primary objective of this trial is to demonstrate that the eGOS 6 months after injury is improved following administration of Ronopterin compared to a placebo control. Exploratory secondary and adjusted multivariable analyses will also be conducted.

### Trial population and eligibility

A total of 220 evaluable patients with moderate or severe TBI will be enrolled in 31 centres with experience in TBI in France, UK, Spain, Austria, and Germany (Appendix 1). The inclusion and exclusion criteria have been chosen to exclude patients with terminal injuries and patients at risk of renal dysfunction.

Patients can be enrolled to the trial if all of the following criteria apply:

1. Written informed consent from the patient’s legal guardian or legal representative or deferred consent procedure, according to local requirements
2. Age 18–60 years, inclusive
3. Expected to survive more than 24 h after admission
4. TBI within the last 18 h (infusion must not start earlier than 6 h after the injury)
5. TBI with Glasgow Coma Score ≥ 3 requiring intracranial pressure (ICP) monitoring
6. Catheter placement (intraventricular or intraparenchymal only) for monitoring and management of increased ICP
7. Systolic blood pressure ≥ 100 mmHg
8. Women of child-bearing potential must have a negative pregnancy test

Patients are excluded if any of the following criteria apply:

1. Penetrating head injury (e.g. missile, stab wound)
2. Concurrent, but not pre-existing, spinal cord injury
3. Bilateral fixed and dilated pupil (> 4 mm)
4. Cardiopulmonary resuscitation performed post injury, or extracranial injuries causing continuing bleeding likely to require multiple transfusions (> 4 units of red blood cells)
5. Coma due to an exclusive epidural haematoma (lucid interval and absence of structural brain damage on computer tomography (CT))
6. Coma suspected to be primarily due to causes other than head injury (e.g. drug overdose intoxication, drowning/near drowning)
7. Known or CT evidence of pre-existing major cerebral damage
8. Patients who cannot be monitored on their recovery (using the eGOS and QOLIBRI)
9. Patients and relatives of patients who do not understand/speak Spanish, English, French, or German
10. Decompressive craniectomy planned prior to randomisation
11. Patients with polytrauma and non-head Injury Severity Score > 18
12. Rhabdomyolysis with serum creatine kinase (CK) > 5000 IU/L
13. Injuries to the ascending aorta and/or carotid arteries and vertebral arteries
14. Serum creatinine > 1.2 mg/dL (106 μmol/L) in women or > 1.5 mg/dL (133 μmol/L) in men
15. Estimated glomerular filtration rate (eGFR) < 60 mL/min
16. Body mass index (BMI) < 18.5 kg/m^2^ and > 40 kg/m^2^, body weight (b.w.) > 110 kg
17. Any severe concomitant condition (cancer; hematologic, renal, hepatic; coronary disease; major psychiatric disorder; chronic alcohol or drug abuse) that can be ascertained at admission
18. Known to have received an experimental drug within 4 weeks prior to current injury

The exclusion criteria are designed to exclude patients with previous kidney damage and patients who cannot be followed up for 6 months.

### Management of traumatic brain injury

The study centres will treat patients according to standard care; however, the centres are requested to follow standardised TBI clinical practice according to current guidelines [12].

### Randomisation

Patients in the trial treatment are allocated to treatment with Ronopterin or placebo in a ratio of 1:1, via a confidential interactive web response system and block randomisation. Balance in treatment allocation across the study participants is enhanced through stratification by both research site (defined by each participating hospital) and by the age of the patient included (two age groups, 18–39 and 40–60 years).

Patients aged 18–39 years represent the group of patients with generally better recovery perspectives than the group of patients aged 40–60 years. The upper age limit was set to 60 years for safety reasons, as in the NOSTRA phase II trial in particular, elderly patients exhibited renal dysfunction [9].

### Trial interventions

The intervention is the intravenous administration of Ronopterin compared to a placebo (0.9% saline) via central venous catheter. A total dose of 17 mg/kg b.w. of Ronopterin is infused over 48 h (daily dose 8.5 mg/kg b.w.). The study drug or placebo is administered over 48 h at the earliest 6 h after injury and at the latest 18 h after the injury.

In the case that serum creatinine values increase by more than 50% from the preceding measurement, the infusion has to be stopped. Any other renal safety parameters that indicate renal deterioration may also cause the infusion to be stopped.

**Fig. 1:**
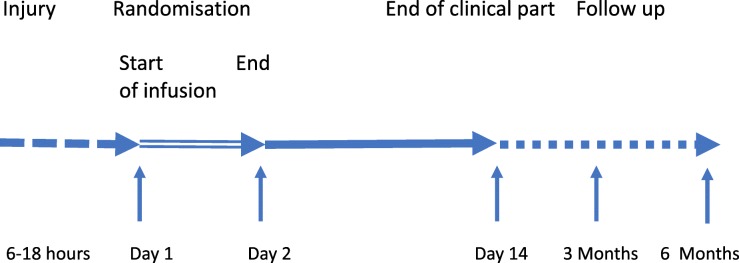
Summary scheme of NOSTRA trial treatment

The administration period is followed by a clinical follow-up period of 14 days. Patients are closely monitored in the first three days after the end of the infusion. The clinical period is followed by a post-clinical period with visits at 3 and 6 months (Fig. 1).

### Investigational medicinal product

Ronopterin (4-amino-tetrahydrobiopterin, VAS203) is provided as a lyophilizate (1 g/vial). The vials are reconstituted with 50 mL of water at the site by a trained unblinded person, to provide a ready-to-use solution. The final concentration of the drug substance Ronopterin is 20 mg/mL. Two vials are used for each patient (one vial for each treatment day).

The doses for each individual patient are calculated automatically according to the individual body weight, using information from the electronic case report form (eCRF). The calculation results in an individual infusion rate for each patient.

### Treatment masking (blinding)

The trial is conducted as a double-blinded trial. Patients, site investigators, site research coordinators, the Sponsor, central CT scan assessor and the staff in charge of treating the patients will not know the treatment allocation. The infusion solution is prepared by unblinded staff at each centre, who are not involved in the care of trial patients. Depending on the local organisation of the site this can be a pharmacist or a nurse from another ward or from the central pharmacy. The unblinded team passes the ready-to-use Ronopterin solution or placebo (masked in opaque orange syringes), labelled with a random number, patient number and infusion rate, to the blinded team. Emergency unblinding can be done via the interactive web response system or - in the case of problems - by the central pharmacy.

### Data collection

Data on all patients (including excluded patients) will be collected by trained study nurses using a web-based eCRF. Queries are generated automatically or by the clinical research associate. Monitoring is performed by the clinical research organisation and the Sponsor (Appendix 2).

Assessments (eGOS and QOLIBRI) for the 6-month outcome will be performed by the investigators by personal interview at the respective centre or by the investigator visiting the patient. All assessors of the eGOS are trained personally (preferred) or via web-based training.

All data and other information generated will be held in strict confidence. The patients will be identifiable only by their patient number. All documents that identify the patient (e.g. informed consent) are maintained in confidence by the investigator.

The Standard Protocol Items: Recommendation for Interventional Trials (SPIRIT) reporting guidelines are applied [13]. Results of the study will be communicated to participants, healthcare professionals and the public by publication and reporting in clinical trial databases (EudraCT, NCT) without any restrictions.

### Study objectives and endpoints

#### Sample size estimation

The main primary and secondary analyses will follow a modified intention-to-treat approach to define the full patient analysis set, based on all randomly assigned patients except those withdrawing consent for use of all trial data and those not fulfilling inclusion criteria and never receiving the intervention.

#### Primary outcome

The primary outcome is the difference between eGOS in patients allocated to Ronopterin and patients allocated to placebo at 6 months after the injury. The eGOS value is determined by a face-to-face meeting; telephone interviews are not planned. Face-to-face meetings allow more accurate and reliable evaluation of the eGOS.

##### Secondary outcomes and pre-specified covariates

Secondary efficacy endpoints are evaluated according to the following priority:

1. Quality of Life after Brain injury (QOLIBRI) score at 6 months after TBI
2. QOLIBRI overall scale (QOLIBRI-OS) score at 6 months after TBI
3. eGOS at 3 months after TBI
4. QOLIBRI-OS score at 3 months after TBI
5. Therapy intensity level (TIL) at 24 h over 14 days after TBI
6. Number of craniectomies (one or both hemispheres)

Further efficacy endpoints for efficacy are:

1. Mortality at 6 months after TBI
2. Intracranial pressure (ICP) and cerebral perfusion pressure (CPP) over 5 days after TBI
3. Individual domains of QOLIBRI
4. Tissue oxygen pressure (PtiO_2_) in cerebral tissue (in centres with respective equipment)
5. Cerebral extracellular glucose, lactate, pyruvate by microdialysis (in study centres with respective microdialysis equipment) on an hourly basis over 5 days. Additionally, glycerol, urea and glutamate in study centres with respective multi-channel microdialysis equipment
6. Prognostic factor

### Sample size and power

In the NOSTRA phase II study, an odds ratio for the eGOS of 25.2 in favour of Ronopterin was estimated using ordinal logistic regression with a lower (two-sided) 95% confidence limit of 3.2 [9]. For the sample size estimation in the current NOSTRA phase III study, an odds ratio of 2.3 was assumed, which can be considered as very conservative compared to the preceding estimates. In addition, the following distribution of eGOS relative occurrences across the levels for patients in the placebo group was assumed starting from level 1 to level 8: 0.09, 0.1, 0.1, 0.15, 0.3, 0.25, 0.005 and 0.005. This is a smoothed distribution reflecting the actual distribution in the placebo group in the NOSTRA II study. Sensitivity considerations have shown that the sample size estimation is rather robust against deviations from the aforementioned assumed distribution in the placebo group. In a simpler data situation dichotomized as 50% of the patients on placebo, an odds ratio of 2.3 approximately corresponds to an increase of favourable responses under treatment of 20%.

In the present study eGOS is also the primary parameter of analysis; randomisation was planned in a patient number ratio of 1:1 to achieve maximum statistical power. It was assumed that the treatment effect of Ronopterin compared to placebo would be tested on a two-sided alpha level of 0.05 using the non-parametric Wilcoxon rank-sum test for ordered categorical data. Derived from the NOSTRA phase II trial, the phase II/III trial was designed to detect a treatment effect of an odds ratio of 2.3 (approximately 10-fold lower as in the phase II trial) as statistically significant on an alpha level of 0.05 with statistical power slightly > 90% (92.2%); 220 evaluable patients are needed for the statistical intention-to-treat analysis in the full analysis set (FAS). With this number of 220 patients a lower odds ratio of 2.0 would still be detected with 80% statistical power (alpha = 0.05, two-sided) while a higher odds ratio of 2.6 could be detected with 90% power even at a two-sided alpha level of 0.01. To account for withdrawals and patients lost to follow up, the number of patients to recruit has been increased by 5% to 232 patients.

These conservative assumptions on the odds ratio are not unreasonably low, because the treatment effect may be diluted by premature infusion terminations and by missing eGOS values on follow up; such protocol violators are included in the FAS but excluded from the per-protocol set. Under favourable conditions the study has a good chance to demonstrate statistical significance even at the lower alpha level of 0.01 and thus to deliver strong statistical evidence of efficacy.

### Statistical analysis

The null hypothesis of no shift across the eight ordered categories of eGOS for the two treatment groups will be tested based on a proportional odds model stratified by age (18–39 years and 40–60 years). The treatment effect will be estimated using ordinal logistic regression as the (proportional) odds ratio of Ronopterin versus placebo with a two-sided Wald 95% confidence interval. Treatment and age (18–39 years and 40–60 years) will be included in the model. The proportional odds assumption will be tested using the chi-square score test. If the proportional odds assumption is not be fulfilled then the individual cumulative odds ratios will be presented with their 95%-confidence intervals.

The interim analysis is to be conducted after half the patients have completed their 6-month assessment (110 patients in the FAS). The proportional odds model stratified by age is used to compare the groups and the associated *p* value, *p*_1_ *p*_1_ is calculated. If the study continues to the end, the proportional odds model stratified by age based on data from patients randomised in the second stage is used to compare the groups and the associated *p* value, *p*_2_ is calculated. According to Bauer and Koehne [14] and Lehmacher and Wassmer [15], the *p* values are combined to control the type I error. If *p*(*p*_1_*p*_2_) is < 0.025, then significance can be claimed.

The efficacy of Ronopterin will be considered to be proven if the null hypothesis for the primary endpoint is rejected and if the treatment difference is in favour of Ronopterin in the sense of a shift to higher eGOS categories under Ronopterin.

### Secondary efficacy analysis

The eGOS variable at 3 months will be analysed using ordinal logistic regression as the (proportional) odds ratio of Ronopterin versus placebo with a two-sided Wald 95% confidence interval. Treatment and age (18–39 years and 40–60 years) will be included in the model. The proportional odds assumption will be tested using the chi-square score test.

The Quality of Life after Brain Injury Index (QOLIBRI) [16] score will be reported at 6 months after TBI. The QOLIBRI overall scale (QOLIBRI-OS) score will be reported at 3 and 6 months after TBI. The total score on the QOLIBRI and QOLIBRI OS will be analysed by analysis of variance (ANOVA) including treatment, age category (18–39 years and 40–60 years) and treatment-by-age interaction as covariates. The adjusted mean difference in the total scores under Ronopterin and placebo, with the 95% confidence interval, will be used as the treatment effect estimate.

The therapy intensity level (TIL) as a measure of intensity of treatment [17] from day 1 to day 14 post trauma will be analysed using a mixed model for repeated measures including treatment, age category (18–39 years and 40–60 years), days, treatment-by-age and treatment-by-day interaction as fixed effects and patient as a random effect. Using these methods, TIL will be analysed across the whole observation period from day 1 to day 14. Treatment effect estimates will be based on adjusted mean differences including 95% confidence intervals.

The number of craniectomies (one or both hemispheres) will be analysed using a generalized linear model - Poisson as the distribution, with treatment, age category (18–39 years and 40–60 years) and treatment-by-age interaction as factor. The estimated mean difference of the natural logarithms with the 95% confidence interval for the mean difference will be used to estimate the treatment effect. The ratio of the mean number of craniectomies per day and its 95% confidence interval can be estimated by transforming the aforementioned estimated mean difference and its 95% confidence interval by the exponential function.

### Further efficacy analysis

Tertiary endpoints will be delineated descriptively and/or tested two-sided in the sense of exploratory data analysis; there will be no alpha correction for these endpoints. The proportions of patients who die will be compared at 6 months after TBI. The estimate of the difference in proportions (Ronopterin versus Placebo), 95% confidence interval and Chi-square *p* value will be calculated. In the case of lower cell frequencies (< 5), the Fisher exact test will be used instead.

Overall survival will be analyzed by log-rank test. Kaplan-Meier estimates of the 25th, 50th and 75th quartiles will be reported. The 95% confidence interval for the median and *p* values will also be calculated.

Absolute values of ICP and CPP (until day 5) will be summarized per treatment group and for each time point by means of descriptive statistics. The 95% confidence intervals will be calculated for the median using non-parametric methods for order statistics.

The comprehensive QOLIBRI assesses health-related quality of life (QoL) within six domains (self-cognition, cognition, daily life and autonomy, social relationships, emotions and physical problems). Total scores in each of these domains of QOLIBRI will be analysed using the *t* test with a two-sided alpha level of 0.05. The mean difference in the scores and the 95% confidence interval under Ronopterin and placebo will be used as the treatment effect estimate.

Partial brain oxygen pressures are recorded every 1 h up to 5 days after the start of the infusion. Absolute values will be summarized per treatment group and for each time point by means of descriptive statistics. The 95% confidence intervals for the mean will be calculated (normal approximation).

The cerebral glucose, lactate, pyruvate and glutamate are recorded every 1 h up to 5 days after the start of the infusion. Absolute values will be summarized per treatment group and for each time point by means of descriptive statistics. The 95% confidence intervals for the mean will be calculated (normal approximation).

**Fig. 2:**
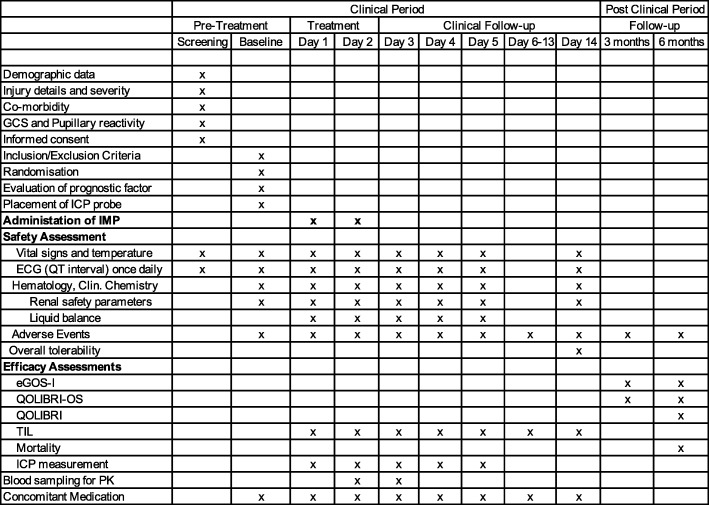
SPIRIT scheme of study procedures

The prognostic factor according to Steyerberg et al. [18] predicts the probability of 6-month mortality or probability of 6-month unfavorable outcome. The prognostic factor will be analysed using the *t* test with a two-sided alpha level of 0.05. The mean difference in the score and its 95% confidence interval under Ronopterin and placebo will be summarized for comparability at baseline. A SPIRIT scheme of all study procedures is shown in Fig. 2 (Additional file 1).

### Data monitoring and interim analyses

An independent Data and Safety Monitoring Committee (DMC) monitors the quality of the trial and has access to trial outcome and accumulated safety data, including serious adverse events (SAEs), suspected unexpected serious adverse reactions and mortality. In addition, the DMC will review the safety data from a clinical and safety point of view on an on-going basis (Appendix 4).

Three safety interim analyses will be scheduled: when 40, 80 and 110 patients, respectively, have completed their 14-day clinical observation phase. When 110 evaluable patients in the FAS have received their final assessment of clinical outcome (6-month eGOS), an unblinded interim analysis will be conducted by an unblinded statistician and reviewed by the DMC based on clean data on the primary and secondary target variables and on the latest status of safety data. The outcome of this interim analysis will result in one of three possible recommendations of the DMC to the Sponsor to do one of the following:

- Stop the study because of futility
- Continue and finalize the study as planned
- Continue the study as planned but increase the sample size to a specified number of patients

There is no intention to stop the study in the interim if the data already show significant outcome differences between Ronopterin and placebo. An increase in sample size will be recommended by the DMC guided by conditional power of 90%, based on the current estimate of the treatment effect. The maximum sample size to be considered is 330 patients.

### Safety and adverse event analyses

Safety analyses will be based on the safety set and will comprise standard descriptive methods. Results of all safety measurements will be summarized by treatment and by pre-treatment period, treatment period, clinical follow-up period (days 3–14) and post clinical follow-up period (3 and 6 months) and across periods overall except for the pre-treatment period.

Descriptive statistics will be calculated for laboratory measurements, vital signs including body weight and body temperature, QT interval, P_t_iO_2_ and renal safety monitoring. Changes from baseline will be summarized using standard statistical characteristics and shift tables. Frequencies of normal, clinically significant abnormal and not clinically significant abnormal findings will be presented overall and for each observation period as indicated above.

The total number of treatment-emergent adverse events (TEAE) and the total number of patients with TEAEs, the total number of TEAEs related to the study drug (certain, probable, possible), the total number of patients with TEAEs related to the study drug, the total number of TEAEs and the total number of patients with serious TEAEs, the total number of patients with TEAEs leading to discontinuation of study treatment and the total number of patients with TEAEs leading to death will be summarized by treatment arm. In addition, adverse event severity, relationship to the study drug, actions taken and other action taken will be summarized.

### Trial status

The effective study protocol is version 14.0 from 5 November 2018. The first patient was enrolled on 25 August 2016. Two interim safety analyses were conducted resulting in approval by the DMC to continue the trial without alteration to the research protocol. The target recruitment will be achieved by the end of 2019, making final 6-month eGOS outcomes available by mid-2020.

## Conclusions

TBI is a severe condition with no specific pharmaceutical therapies available. The administration of Ronopterin has the potential to improve clinical outcome. The NOSTRA-III trial aims to detect a beneficial effect of Ronopterin on clinical outcomes after TBI or to provide the basis for an additional pivotal clinical trial, while minimising any potential risk, in particular for renal function.

### Supplementary information

**Additional file 1.** Reporting checklist for protocol of a clinical trial based on the SPIRIT guidelines.

## Data Availability

Not applicable.

## Appendix 1

### NOSTRA-Trial Principal and Deputy Investigators

Medizinische Universitaet Innsbruck, Austria: Ronny Beer (PI), Raimund Helbok, Mario Kofler, Bettina Pfausler, Alois Schiefecker.

LKH – Universitaetsklinikum Graz, Austria: Michael Mokry (PI), Etienne Holl, Amir Mohia, Karin Pistracher, Stefan Riegler, Frank Unger, Sonja Wissa.

Medizinische Universitat Wien, Austria: Harald Willschke (PI), Johannes Leitgeb, Martin Niederle, Rupert Schuster,

Hopital Gabriel Montpied Clermont-Ferrand, France: Russell Chabanne (PI), Thibaud Cammas, Bernard Cosserant, Kevin Lagarde.

Hopital Pellegrin Bordeaux France: Vincent Cottenceau PI), Cedric Carrie, Melanie Lafitte, Laurent Petit.

Hopital Universitaire Caremeau Nîmes France: Jean-Yves LeFrant (PI), Florian Bazalgette, Stephanie Bulyez, Aurelien Daurat, Ghislaine Gardes, Guillaume Louart, Pablo Massanet, Laurent Muller, Claire Roger, Gilbert Saissi, Remi Trusson,

HIA Sainte Anne Toulon, France: Ambroise Montcriol (PI), Claire Contargyris, Pierre Esnaut.

Heinrich-Heine-Universitaet, Düsseldorf, Germany: Kerim Beseoglu (PI), Rainer Kram.

Universitätsklinikum Schleswig-Holstein Kiel, Germany: Michael Synowitz (PI), Charlotte Flueh.

Berufsgenossenschaftliches Universitaetsklinikum Bergmannsheil Bochum, Germany: Ramon Martinez-Olivera (PI), Bogdan Pintea.

BG Klinikum Bergmannstrost Halle Germany: Hans-Joerg Meisel (PI), Stefan Bone, Christian Glien, Axel Grossstueck, Bodo Kern, Rene Koch, Sebastian Langer, Chida Rajendran, Peter Stosberg, Julia Tews.

Universitätsklinikum Leipzig, Germany: Juergen Meixensberger (PI), Felix Arlt, Dirk Lindner.

Allgemeines Krankenhaus Celle, Germany: Eckhard Rickels (PI), Mihai Bercea, Boyan Ivanov.

Universitätsmedizin Goettingen, Germany: Veit Rohde (PI), Christoph Bettag, Dorothee Mielke, Christina Wolfert.

Universitätsklinikum Frankfurt, Germany: Christian Senft (PI), Elisabeth Adam, Bedjan Behmanesh, Markus Bruder, Daniel Dubinski, Florian Gessler, Fatma Kilinc, Stephanie Wallenwein, Sae-Yeon Won.

Universitätsklinikum des Saarlandes Homburg / Saar, Germany: Jacek Szczygielski (PI), Akos Csokonay, Doerthe Keiner, Dorothea Anna, Maria Muenchen.

Universitätsklinikum Heidelberg, Germany: Andreas Unterberg (PI), Martin Grutza, Mohammed Nofal, Bilal Younes, Alexander Younsi, Klaus Zweckberger.

Universitätsklinikum Jena, Germany: Jan Walter (PI), Kristina Decheva, Denise Feierabend.

University Medical Center Eppendorf Hamburg, Germany: Manfred Westphal (PI), Patrick Czorlich, Malte Mohme, Thomas Sauvigny, Miriam Schaper,

Medizinische Hochschule Hannover, Germany: Florian Wild (PI), Majid Esmaeilzadeh Moghaddam, Felix Kiepe, Josef Lang.

Charite Campus Virchow Berlin, Germany: Stefan Wolf (PI), Tobias Finger, Nils Hecht, Lars Wessels.

Espases University Hospital Palma de Mallorca, Spain: Javier Ibanez (PI), Daniel Alegre Ruano, Marta Brell, Santiago Garfias, Victor Goliney, Adriana Gomez-Martin, Victor Gonzalez-Jimenez, Paloma Jimenez-Fernandez, Monica Lara, Juan Antonio Llompart, Sofia Lopez-Lage, Milton Martinez, Antonio Mas, Apolonia Moll, Lesmes Moratinos, Juan Perez, Maria Picado, Sergio Rocabado, Osman-Alberto Salazar-Asencio, Julio Velasco.

Hospital Universitario 12 de Octubre Madrid, Spain: Alfonso Lagares (PI), Ana Castano-Leon, Santiago Cepeda-Chaflan, Mario Chico-Fernandez, Carla Eiriz-Fernandez, Jose Fernandez-Alen, Daniel Garcia- Perez, Pedro Gomez-Lopez, Pedro Gonzalez-Leon, Luis Jimenez-Roldan, Pablo Martin-Munarriz, Luis Moreno-Gomez, Carolina Mudarra, Irene Panero-Perez, Igor Paredes-Sansinenea, Angel Perez-Nunez.

Hospital Universitario ELX Elche-Alicante, Spain: Ana Perez (PI), Michael Bartz, Raquel Flores, Francisco Martinez-Penuela, Natividad Mas-Veracruz, Maria Mercader, Jaime Miralles.

Vall d’Hebron University Hospital Barcelona, Spain: Joan Sahuquillo (PI), Fuat Arikan, Jacinto Baena, Marcelino Baguena, Jose Mauricio Cevallos-Calero, Dario Gandara-Sabatini, Diego Lopez, Francisco Ramon Martinez-Ricarte, Maria Antonia Poca, Marilyn Riveiro,

Western General Hospital Lothian University Edinburgh, UK: Peter Andrews (PI), John Emelifeonwu, Jonathan Rhodes.

Queen Elizabeth Hospital Birmingham, UK: Antonio Belli (PI), David Davies, Catherine Snelson, Tonny Veenith, Tony Whitehouse, Kamal Yakoub.

Wessex Neurological Centre University Hospital Southampton, UK: Diederik Bulters, Mukul Arora, Patrick Holton, Silvester Kabwama, Rossana Romani, Ardalan Zolnourian.

Kings College Hospital London, UK: Christos Tolias (PI), Prajwal Ghimire, Philip Hopkins

## Appendix 2

### NOSTRA-trial management

Ingrid Pfeuffer and Claudia Wolf Werner, vasopharm GmbH, Germany: Clinical trial management.

Lindsay Wilson, Division of Psychology, University of Stirling, UK: eGOS training.

Henry Boret, Centre Hospitalier Intercommunal Toulon, La Seyne-sur-Mer France: Central Review of CTs

## Appendix 3

### List of Central Ethics Committees

Austria: Ethikkommission Med. Universität Innsbruck; date of approval 31 May 2016

France: Comite de protection des personnes sud mediterranee IIII, Nimes; Date of approval 09May2016.

Germany: Ethikkommission der Med. Fakultät Heidelberg; Date of approval 15Nov2016.

Spain: Comite de etica de la investigacion con medicamentos del Universitario Vall d’Hebron.

Barcelona; Date of approval 03Jun2016.

UK: South Hampshire A Research Ethics Committee Bristol; Date of approval 23May2016

## Appendix 4

### NOSTRA-Trial DMC

Prof. Dr. med. Thomas Gramatté Drug Development Consulting, Dresden, Germany.

Prof. Dr. med. John F. Stover; Zuerich, Switzerland.

Prof. Dr. med. Roland Schmieder Friedrich-Alexander-Universität Medizinische Klinik, Germany.

Dr. Winfried Koch (Statistics) HaaPACS GmbH, Schriesheim, Germany.

## Abbreviations

b.w.: Body weight
CRO: Clinical Research Organisation
CT: Computer tomography
DMC: Data and Safety Monitoring Committee
eCRF: Electronic case report form
eGOS: Extended Glasgow Outcome Score
FAS: Full analysis set
ICP: Intracranial pressure
PtiO_2_: Tissue oxygen pressure
QOLIBRI: Quality of Life after Brain injury Index
SAE: Severe adverse event
TBI: Traumatic brain injury
TEAE: Treatment-emergent adverse events
TIL: Therapy intensity level

## References

[1] Stocchetti N, Carbonara M, Citerio G, Ercole A, Skrifvars MB, Smielewski P, Zoerle T, Menon DK (2017). Severe traumatic brain injury: targeted management in the intensive care unit. Lancet Neurol.

[2] Maas AI, Peeters W, van den Brande R, Polinder S, Brazinova A, Steyerberg EW, Lingsma HF (2015). Epidemiology of traumatic brain injury in Europe. Acta Neurochir (Wien).

[3] Maas AIR, Menon DK, Adelson PD, Andelic N, Bell MJ, Belli A (2017). InTBIR Participants and Investigators. Traumatic brain injury: integrated approaches to improve prevention, clinical care, and research. Lancet Neurol.

[4] Bragge P, Synnot A, Maas AI, Menon DK, Cooper DJ, Rosenfeld JV, Gruen RL (2016). A state-of-the-science overview of randomized controlled trials evaluating acute management of moderate-to-severe traumatic brain injury. J Neurotrauma.

[5] Cherian L, Hlatky R, Robertson CS (2004). Nitric oxide in traumatic brain injury. Brain Pathol.

[6] Schinzel R, Tegtmeier F, Kim Heidenreich K (2017). Nitric oxide synthase inhibition in traumatic brain injury. New therapeutics in TBI.

[7] Terpolilli NA, Zweckberger K, Trabold R, Schilling L, Schinzel R, Tegtmeier F, Plesnila N (2009). The novel nitric oxide synthase inhibitor 4-amino-tetrahydro-L- biopterine prevents brain edema formation and intracranial hypertension following traumatic brain injury in mice. J Neurotrauma.

[8] Schwarzmaier SM, Terpolilli NA, Dienel A, Gallozzi M, Schinzel R, Tegtmeier F, Plesnila N (2015). Endothelial nitric oxide synthase mediates arteriolar vasodilatation after traumatic brain injury in mice. J Neurotrauma.

[9] Stover JF, Belli A, Boret H, Bulters D, Sahuquillo J, Schmutzhard E (2014). NOSTRA-II Investigators. Nitric oxide synthase inhibition with the antipterin VAS203 improves outcome in traumatic brain injury: a placebo-controlled randomized Phase IIa trial (NOSTRA). J Neurotrauma.

[10] Ott C, Bosch A, Winzer N, Friedrich S, Schinzel R, Tegtmeier F, Schmieder RE (2019). Effects of the nitric oxide synthase inhibitor ronopterin (VAS203) on renal function in healthy volunteers. Br J Clin Pharmacol.

[11] McMillan T, Wilson L, Ponsford J, Levin H, Teasdale G, Bond M (2016). The Glasgow Outcome Scale - 40 years of application and refinement. Nat Rev Neurol.

[12] Carney N, Totten AM, O'Reilly C, Ullman JS, Hawryluk GW, Bell MJ, Bratton SL, Chesnut R, Harris OA, Kissoon N, Rubiano AM, Shutter L, Tasker RC, Vavilala MS, Wilberger J, Wright DW, Ghajar J (2017). Guidelines for the Management of Severe Traumatic Brain Injury, Fourth Edition. Neurosurgery.

[13] Chan A-W, Tetzlaff JM, Altman DG, Laupacis A, Gøtzsche PC, Krleža-Jerić K, Hróbjartsson A, Mann H, Dickersin K, Berlin J, Doré C, Parulekar W, Summerskill W, Groves T, Schulz K, Sox H, Rockhold FW, Rennie D, Moher D (2013). SPIRIT 2013 statement: defining standard protocol items for clinical trials. Ann Intern Med.

[14] Bauer P, Köhne K (1994). Evaluation of experiments with adaptive interim analyses. Biometrics..

[15] Lehmacher W, Wassmer G (1999). Adaptive sample size calculations in group sequential trials. Biometrics..

[16] von Steinbüchel N, Wilson L, Gibbons H, Hawthorne G, Höfer S, Schmidt S, Bullinger M, Maas A, Neugebauer E, Powell J, von Wild K, Zitnay G, Bakx W, Christensen AL, Koskinen S, Sarajuuri J, Formisano R, Sasse N, Truelle JL (2010). QOLIBRI Task Force. Quality of Life after Brain Injury (QOLIBRI): scale development and metric properties. J Neurotrauma.

[17] Maas AI, Harrison-Felix CL, Menon D, Adelson PD, Balkin T, Bullock R, Engel DC, Gordon W, Langlois-Orman J, Lew HL, Robertson C, Temkin N, Valadka A, Verfaellie M, Wainwright M, Wright DW, Schwab (2011). Standardizing data collection in traumatic brain injury. J. Neurotrauma.

[18] Steyerberg EW, Mushkudiani N, Perel P, Butcher I, Lu J, McHugh GS, Murray GD, Marmarou A, Roberts I, Habbema JD, Maas AI (2008). Predicting outcome after traumatic brain injury. PLoS Med.

