# Correction to: Efficacy of Ronopterin (VAS203) in Patients with Moderate and Severe Traumatic Brain Injury (NOSTRA phase III trial): study protocol of a confirmatory, placebocontrolled, randomised, double blind, multi-centre study

**DOI:** 10.1186/s13063-020-4107-8

**Published:** 2020-02-12

**Authors:** Frank Tegtmeier, Reinhard Schinzel, Ronny Beer, Diederik Bulters, Jean-Yves LeFrant, Joan Sahuquillo, Andreas Unterberg, Peter Andrews, Antonio Belli, Javier Ibanez, Alfonso Lagares, Michael Mokry, Harald Willschke, Charlotte Flüh, Erich Schmutzhard, on behalf of the NOSTRA Investigators

**Affiliations:** 1 vasopharm GmbH, Würzburg, Germany; 2 Medizinische Universität Innsbruck, Innsbruck, Austria; 3 Wessex Neurological Centre University Hospital, Southampton, UK; 4 Hopital Universitaire Caremeau, Nimes, France; 5 Vall d’Hebron University Hospital, Barcelona, Spain; 6 Universitätsklinikum Heidelberg, Heidelberg, Germany; 7 Western General Hospital Lothian University, Edinburgh, UK; 8 Queen Elizabeth Hospital, Birmingham, UK; 9 Espases University Hospital, Palma de Mallorca, Spain; 10 Hospital Universitario 12 de Octubre, Madrid, Spain; 11 LKH – Universitätsklinikum Graz, Graz, Austria; 12 Medizinische Universität Wien, Wien, Austria; 13 Universitätsklinikum Schleswig-Holstein, Kiel, Germany

**Correction to: Trials (2020) 21:80**

**https://doi.org/10.1186/s13063-019-3965-4**

After publication of our article [1] the authors have notified us that one of the names has been incorrectly spelled.

- Original name spelling:
  Charlotte Flühe
- Correct name spelling:
  Charlotte Flüh

The original article has been corrected.

## References

[1] Tegtmeier (2020). Efficacy of Ronopterin (VAS203) in Patients with Moderate and Severe Traumatic Brain Injury (NOSTRA phase III trial): study protocol of a confirmatory, placebocontrolled, randomised, double blind, multi-centre study. Trials.

